# Developing Food Consumer Attitudes towards Ionizing Radiation and Genetic Modification

**DOI:** 10.3390/nu16203427

**Published:** 2024-10-10

**Authors:** Iwan Junaedi, Lisa S. McNeill, Robert P. Hamlin

**Affiliations:** Department of Marketing, University of Otago, Dunedin 9054, New Zealand; iwan.junaedi@gmail.com (I.J.); rob.hamlin@otago.ac.nz (R.P.H.)

**Keywords:** ionizing radiation, irradiation, food irradiation, irradiated food, genetically modified food, perishable food, food technology neophobia, supply chain, sustainable living

## Abstract

Background/Objectives: This study investigates consumer perceptions and acceptance of ionizing radiation (IoR) as a perishable food stabilisation technology. Consumers’ preferences influence the success of emerging food technologies. Therefore, a comprehensive understanding of consumers’ behavioural responses and their development over time is essential. Methods: This research employs a mixed-methods approach, surveying 313 young adults in New Zealand on their views of both irradiated (IoR) and genetically modified (GM) highly perishable foods. This study explored both participants’ attitudes towards these two technologies and also their willingness to consume these foods. Results: The qualitative research revealed a preponderance of “affective” associations over “cognitive” associations with regard to both IoR and GM technologies. The quantitative research indicated that where consumers were given time to reflect, evaluations of GM improved, while those of IoR did not (*p* < 0.01). There was a gender divide, with females being more positively inclined towards GM and males towards IoR (*p* < 0.01). Both technologies were significantly disfavoured compared to non-treated products (*p* < 0.01). There was a significant discrimination when the two technologies were presented as concepts and as products. GM was more favourably received as a concept than as a product (*p* < 0.01), while IoR was disfavoured in either form. The two food neophobia scales that were tested showed a divergence in performance, with the more affectively based scale showing a higher level of correlation with behaviour. Conclusions: This research reveals that a largely affective (visceral) distrust of both IoR and GM exists within this young food consumer sample. As it is affective in nature, this position will be very resistant to education efforts, particularly if they are “cognitively” based. However, a significant softening of these affective attitudes towards GM products indicates that such efforts may be effective, given time and investment.

## 1. Introduction

Within perishable food supply chains, effective quality management is critical, particularly for highly perishable provisions like fruit and vegetables, whose qualities can rapidly deteriorate if conditions are suboptimal [1,2,3]. The complex collaboration between suppliers, manufacturers, warehouses, and stores within the supply chain aims to optimize production and distribution while meeting quality expectations and timelines [4]. This complexity is amplified for highly perishable foods due to intrinsic and extrinsic factors influencing degradation, ranging from metabolic processes post-harvest to the impact of temperature and atmospheric conditions [5,6]. Losses within the highly perishable supply chain are still significant and may increase as new pests and pathogens emerge [7]. Ionizing radiation (IoR) has long been known to be a highly effective tool in highly perishable food preservation, but its adoption has been slowed by a combination of consumer ignorance and suspicion, with a strong connection between these two attitudes [8,9,10,11].

This study investigates consumers’ perceptions and willingness to accept IoR, as it has the potential to significantly reduce highly perishable food wastage [12,13,14]. As attitudes to novel technologies can be changed, it also investigates the relative consumer acceptance of IoR and genetically modified (GM) foods, a technology that has overcome significant and continuing public misgivings to become a widespread tool of food production [15,16].

### 1.1. Ionising Radiation and Fresh Fruit and Vegetables in the Highly Perishable Foods Supply Chain

Fruit and vegetables represent a significant challenge within the food supply chain because they are susceptible to microbial proliferation because of their high water and nutrient content and the loss of natural resistance [6]. Furthermore, unlike some microorganisms, food-borne viruses can endure and affect food at different stages of production from pre- to post-harvest and continue to be infectious to consumers [17]. Traditional thermal treatments used to decontaminate fresh fruit and vegetables cause changes to their physical, nutritional, or bioactive properties and affect their content [18]. In addition, washing and chemical sanitizing treatments create contamination issues and are often ineffective at reducing microbial contamination to ensure consumer safety [19].

As a result, consumers often view these treatments negatively due to concerns about their eating quality, safety, and environmental impact [20]. The need to develop new post-harvest decontamination processes has increased due to the emergence of microorganisms that are more resistant to conventional food preservation techniques [21]. As a result, the industry is exploring novel or non-thermal technologies to address these demands [22]. Several technologies that have been investigated for decontamination include high hydrostatic pressure (HHP), pulsed electrical fields (PEF), ultraviolet irradiation, and IoR [8].

IoR utilizes gamma rays, mainly radioisotopes cobalt-60 and cesium-137 [23], which has gained heightened attention as a potential method to extend the shelf life of fresh fruit and vegetables [24,25,26]. This method eliminates pathogenic microorganisms without causing significant changes to their sensory characteristics [26,27,28]. The Codex Alimentarius released the General Standard for Irradiated Foods in 1983. This standard confirmed all IoR foods’ safety and nutritional sufficiency, asserting they did not present any microbiological concerns. Low radiation doses, ranging from 0.1 kGy to 1 kGy, can hinder germination and delay the ripening process [17,29]. Medium doses, between 1 kGy and 10 kGy, can effectively eliminate pathogenic and spoilage microorganisms [30]. High doses exceeding 10 kGy can be utilized for sterilization and decontamination in various food applications [31]. Due to its controversiality, this study aims to further deduce consumers’ perceptions regarding the underutilized and underexplored IoR technology [32].

### 1.2. Consumer Risk Perceptions and Novel Food Technologies

IoR offers a considerable opportunity to cut waste within the fresh fruit supply chain. However, products treated with novel food technologies, such as PEF and IoR, present complex challenges for researchers investigating the factors influencing eventual consumer preferences [32,33,34,35,36], purchasing behaviour [28], and acceptance [37] of food items that have been subjected to IoR. The primary challenges revolve around the consumer benefits [38], encompassing concerns regarding the potential health impacts, exposure to various chemicals or radioactivity, and the technology’s influence on affordability. The impact of consumers’ perceived risks associated with novel food technologies is evident in the ongoing research and debate surrounding IoR and GMOs [39,40]. While these two technologies receive notable scrutiny from the public and in research, numerous other seemingly less risky food technologies can evoke perceptions of risk and associated concerns [33]. Consumers’ concerns may overshadow the potential benefits of novel technology processes and hinder the market introduction of products utilizing these technologies [33,41,42].

To counteract the adverse perception of foods derived from novel technologies and build trust among consumers, researchers in the food industry are concentrating on public education and spreading information about these food products [43]. Whether the public embraces or rejects IoR food hinges on the availability or absence of information [44]. Information about products, including product names and labels, can influence product recognition, preference, perceived sensory and image attributes, acceptance, intended purchase, and consumption [32,41,45,46,47]. The role of information communicated through labelling is crucial since consumers cannot directly perceive the benefits of novel technologies and foods from the product itself [48]. Moreover, information about IoR benefits on a product label, such as enhanced food safety, boosts consumer acceptance [49].

Irradiation frequently triggers negative associations among consumers, such as thoughts of nuclear disasters, which leads to a lack of acceptance of irradiated food [20,50]. Furthermore, consumers often incorrectly believe that irradiated products can become radioactive, pose environmental risks, and have reduced nutrient content despite scientific evidence to the contrary [8,29,51]. Naturalness holds considerable value regarding food, where natural foods are typically associated with greater healthiness, tastiness, and environmental friendliness [52]. Foods created with minimal human interference are inherently seen as safe [53]. The negative perception of highly processed foods largely stems from their perceived lack of naturalness [51,54].

### 1.3. Consumer Risk Perceptions and Consumer Behaviour Theory

At present, IoR faces its greatest challenges at the consumer level, and these challenges stem from the sub-cognitive and heuristically based evaluation and decision processes that are deployed by consumers when evaluating food [55].

The affect heuristic, a psychological theory, explains how negative associations influence consumers’ views of novel food technologies [41,56]. It suggests that people often rely on emotions [57,58] or mental shortcuts (heuristics) rather than a careful analysis of facts when making decisions [59], because of human cognitive limitations in evaluating risk and uncertainty. This results in the simplification of complex issues to facilitate decision-making [60], like evaluating IoR or GMOs. This theory highlights two challenges in understanding consumers’ views of food radiation: first, they may not realize that foods can be contaminated [45,61], and second, due to this lack of risk awareness, they may rely on emotional associations relating to IoR rather than investing time and attention in assessing food technology methods [62,63].

Correspondingly, the availability heuristic is based on the idea that events with higher probabilities are more readily recalled than unlikely ones, and the associations between events are strengthened through frequent co-occurrence [64]. This phenomenon may explain the difficulties consumers encounter in imagining low-probability or high-consequence events happening to them, resulting in an overestimation of infrequent events and an underestimation of frequent events. Another associated outcome is that a low-probability hazard can increase the perceived likelihood of the hazard, regardless of the actual evidence [50]. This is significant in contexts where new information, such as that regarding a new food technology, is considered by the consumer.

Moreover, variability in people’s preferences and values may contribute to variations in how consumers embrace novel food technologies [65]. Previous studies have focused on factors like the FTN [66,67,68], disgust sensitivity [69], and cultural influences to understand individual differences in risk perception and acceptance. Nevertheless, recent studies have indicated that individuals’ personality traits, such as openness, shape their preferences for IoR and GMOs [51,70].

### 1.4. Food Neophobia (FN) and Food Technology Neophobia (FTN)

FN refers to the apprehension, aversion, or repulsion towards unfamiliar foods [71]. It is influenced by evolutionary factors and, to some extent, moulded by family customs that dictate an individual’s dietary preferences [72]. When viewed in a broader cultural context, these traditions contribute to the development of neophobia [73]. In general, FN is defined as the hesitancy to consume or avoid novel foods [74]. On the other hand, FTN is a personality or psychological trait that impacts consumers’ readiness to embrace new food technologies or adopt innovative methods for producing new foods [51]. The primary elements contributing to consumers’ reluctance to try foods created through new food technologies typically encompass functional barriers linked to the ease of use and utility, perceptions of benefits and risks, knowledge and attitudes, socio-demographic indicators, lifestyle factors, and psychological barriers [75,76].

Thus, the necessity to develop a novel psychometric tool designed to measure FTN, which has been introduced as part of a research initiative on consumer acceptance of innovative foods, known as the Food Technology Neophobia Scale (FTNS) [66]. The FTNS has demonstrated its efficacy as a reliable measure of consumers’ neophobia levels towards foods processed through innovative technologies. For example, a study on food packaging in Canada [77]; FTN towards processed Matooke flour in Uganda [78]; and a study on food processing in Italy [79]. This study aims to enhance the breadth of the applicability of the FTNS to food products generated by innovative technologies like the IoR. By comparing the standard fresh fruit, GM fruit, IoR fruit, and a combination of GM and IoR fruit, this research applied the FTNS to assess the influence of FTN. The fundamental hypothesis driving this study aims to determine whether the apparent preference for IoR food results from consumers’ anxiety towards new technology or the term “radiation”.

In addition to FTNS, word association is employed in numerous studies investigating attitudes towards food [80,81,82]. This method is valuable for swiftly probing consumer perceptions regarding novel and undefined concepts [83]. It excels in capturing the emotional and less conscious aspects of respondents’ thoughts, surpassing more direct questioning methods [84]. In this context, it delves into the associations individuals form with each of the suggested names, shedding light on the role of anchoring in shaping attitudes towards unfamiliar concepts [85]. For example, in one study, food irradiation may evoke connections with nuclear power and radioactivity [25,54,86]. In another study, “food ionization” was favoured over “food irradiation”, even though both terms refer to the same strategy for decontaminating food [41].

### 1.5. Research Questions

While there is extensive literature on general consumer responses to IoR food, a notable gap exists in understanding how consumers associate the presented facts and leverage their existing knowledge to shape their perceptions and intentions regarding IoR food. This research examines how consumers in New Zealand, mainly young and well-educated individuals, respond to IoR food and explore potential alternatives. The rationale behind selecting young consumers lies in their propensity to embrace novel food options and their heightened awareness of health and environmental considerations [87]. Therefore, this demographic offers valuable insights into the attitudes and preferences of those individuals most inclined to explore IoR food and its alternatives.

The social representation theory explains how the public comprehends new and unfamiliar concepts [88]. It is particularly intriguing to reveal how individuals navigate the unfamiliar and how they might comprehend IoR by drawing comparisons with more familiar concepts or technologies. The previous research discovers some indications that individuals anchor IoR to a more established technology, such as GMOs [24], to form their understanding of it. The assessment of any product may encompass implicit and explicit attitudes [89]. Implicit attitudes anticipate automatic reactions, like perceiving the radioactivity of IoR and eliciting a disgust response, which is to be expected. Explicit attitudes can be swayed through effective marketing and the dissemination of information [87]. Promoting the benefits of IoR through positive educational messages has been demonstrated to enhance attitudes towards IoR [49].

However, based on the expectancy–value model that can comprehend the potential mechanisms underlying the effects of the information [33], the application of disconfirmed expectations as an explanatory mechanism suggests that when the information about a product, like a label or other extrinsic information, does not align with what consumers expected, it can lead to changes in their attitudes and subsequent behaviour [35]. This misalignment between expectation and reality can impact how individuals perceive and respond to that product, potentially influencing their preferences and attitudes [18]. Therefore, there is a need for additional research to explore the effects of different product features within the IoR concept description for enhancing communication and marketing strategies when introducing IoR to the public. This study aims to address this gap by investigating the intricacies of consumer attitude formation concerning the IoR concept. Specifically, it aims to address the following questions:

**RQ1.**  *What associations do consumers make with the GMOs and IoR fruit concept in relation to fresh fruit?*

**RQ2.**  *What are the behavioural intentions of consumers towards GMOs and IoR fresh fruit?*

The FTNS has been identified as a reliable tool for evaluating consumer apprehensions related to food technologies due to its specific emphasis on technology rather than food [66]. Individuals with higher scores on the FTNS, indicating greater FTN, are more inclined to reject tasting foods created by novel technologies, especially those considered more controversial [67]. Consumers frequently link IoR with causing radioactivity in food, which is unlikely given the permitted doses for food radiation [90]. Furthermore, the procedure cannot elevate the natural radioactivity level of the food, regardless of the duration of exposure to radiation or the absorbed energy dose. However, while the FTNS remains a reliable gauge of FTN, it is imperative to acknowledge that acceptance or rejection of specific food products may be influenced by variations in consumers’ location, technology, culture, and the nature of the food in consideration [33,68].

**RQ3.**  *Is FTN an effective predictor of consumer attitudes and behavioural intentions towards IoR fruit?*

## 2. Materials and Methods

### 2.1. Survey

This research utilized a survey employing both closed and open-ended response options. This methodology generated a dataset comprising qualitative and codable quantitative data, enabling a detailed investigation of the research questions outlined above. Survey participants were recruited via a direct, personal approach to individuals within the University of Otago campus. Prospective participants confirmed their age of 18 years or older and provided written informed consent to participate in the survey. The surveys were conducted in a “face-to-face” manner using pen and paper. The instrument is shown in Appendix A.

Each interview constituted a substantial face-to-face combined qualitative and quantitative research exercise. The sample size was thus dictated by the quantitative component, and thus, greatly exceeded the “saturation size” requirement of qualitative component [91]. The quantitative sample size was set to achieve the necessary reduction of risk via statistics while using a minimal individual exercise sample size and multiple replications to reduce sample non-homogeneity-related experimental uncertainty [92,93]. A sample of 160 (4 × 40) responses was thus collected for each 2 × 2 factorial experimental exercise, with two replications, yielding a total observation sample of 320 responses, with 7 being incomplete and subsequently excluded from the analysis. As the absolute minimum sample size was 4 × 30 × 2 (240), the seven excluded responses did not affect the validity of the sample.

This resulted in a total of 313 valid responses for analysis. There are four different treatments for each replication: (1) standard fresh fruit, (2) GM fruit, (3) IoR fruit, and (4) fruit that is both GM and IoR. Nearly every individual passing the researchers’ location was invited to participate, with a small courtesy incentive provided (a bar of chocolate). This technically constitutes a convenience sample, as it comprised students present on campus then. There were no exclusion criteria.

### 2.2. Consumer Reactions to and Associations with the GMOs and IoR Concept (RQ1)

RQ1 was a qualitative research exercise in which the participants were able to give open-ended responses as a reaction to “standard” GMO and IoR products. Participants initially engaged in a practice word association task to clarify research tasks without influencing the sample through direct discussions about food technology. In this task, they were presented with the word “smartphone” accompanied by a representative image and instructed to spontaneously provide up to four words, phrases, feelings, or thoughts that came to mind in response to this concept. After completing the word association task, participants were requested to assess their perception of the “smartphone” concept using a 5-point scale, ranging from “highly positive” to “highly negative” [85]. Participants were then familiarized with the concept of fresh fruit, GM fruit, IoR fruit, and GM and IoR fruit through a comparable visual presentation coupled with a neutral written description of the product.

Following this introduction, participants were prompted to provide four words or brief phrases encapsulating their reactions to both the visual and written representations of food technologies. These responses, expressed in open-ended free text, underwent coding for subsequent analysis. The concepts presented were meticulously developed through consultations with researchers and aligned with the existing literature on food technologies. The free-text responses in this study underwent analysis using an open, followed by axial, coding approach to discern key second-order themes derived from the proposed concepts [94]. To initiate the coding process, related meanings and response patterns were identified from the text responses [95]. Coding credibility was ensured through a reflexive researcher triangulation process, wherein two research team members verified the alignment of each set of first-order concepts with their second-order theme [96].

Corpus Linguistics (CL) was used to analyse consumers’ emotional experiences by measuring the association between the GMOs or IoR concept and the responses that prevent consumers from purchasing GMOs or IoR fruit. CL is a subset of linguistics and adopts an empirical methodology in analysing real-world language, extracting patterns from datasets [97]. Words or terms are linked to specific text topics, and weights for term–topic and document–topic are assigned to indicate the extent of association. This method incorporates functional terms such as term frequency in both documents and the corpus, the count of documents in the corpus, the count of (unique) terms in a document or corpus, and the maximum frequency of any term in a document. Additionally, it utilizes functional forms encompassing various operators, including addition, subtraction, multiplication, division, log, and square root functions [98,99].

Significant progress in the theoretical foundations of these weights was achieved as researchers increasingly recognized the relevance of Information Theory to the challenge of the term weighting [100]. An essential characteristic of a valuable and reliable weighting algorithm is its ability to assign low or zero weights to terms or topics that appear infrequently or frequently. This aligns with our human heuristics, as we tend to attribute maximum weight or relevance to information somewhere between these extremes [101]. The SAS Enterprise Miner Workstation 15.2 software is employed to conduct the text topic modelling. Text topic modelling involves several crucial steps [102]: Firstly, after importing the data, it parses them into term grammar roles. Roles with minimal or no information, such as prepositions, are excluded. Secondly, the data are filtered, wherein Information Theory-based term weights are computed following best practices as detailed earlier. Terms with limited informative value, determined by a lower quartile term weight, have their document–topic weight thresholded and set to zero. Lastly, documents receive document–topic weights, with similar thresholding applied to poor document–topic association weights.

Following the completion of direct comparisons between IoR fruit and its alternatives, respondents were prompted to undertake a comprehensive personal evaluation of the GMOs and IoR fruit concept. This evaluation was conducted through a 19-item attitude and behavioural intention scale, utilizing a 5-point Likert scale ranging from “strongly agree” to “strongly disagree”. The 19 dimensions employed in the scale were adapted from prior research. Subsequently, the gathered data were utilized to construct a “phobia profile” specific to IoR fruit for the entire sample. Furthermore, an aggregated “IoR fruit phobia score” was computed for individual respondents, allowing for a direct comparison with individual consumer scores on a related but more generalized FTNS.

### 2.3. Consumer Willingness to Purchase GMOs and IoR Products Relative to Alternative Products (RQ2)

This topic was addressed by a quantitative experiment. A 2 × 2 full factorial “between subjects” design with two replications was used with consumption and purchase intent as the dependent variables. The two axes were GMO/non-GMO and IoR/non-IoR, which generated four treatment conditions for the fruit product that was used: “Standard”, “GMO”, “IoR”, and “GM + IoR”. The data were analysed using a simple analysis of variance and Tukey’s test for the significance of differences in multiple means [103].

Participants were asked to assess their readiness to try GMOs and IoR products, specifically strawberries, using a 5-point Likert scale ranging from “I would definitely purchase” to “I would definitely not purchase”. Subsequently, they were introduced to the concepts of standard fresh strawberry, GM strawberry, IoR strawberry, and GM and IoR strawberry. Participants were asked to rate their willingness to consume these as equivalent strawberry products. This process aimed to establish a comparative measure of willingness to purchase IoR products in relation to other alternative products in the market.

### 2.4. Neophobia as a Predictor of Consumer Attitudes and Behavioural Intention towards IoR Products (RQ3)

Two neophobia scales were tested in this part of the research by comparing the degree of individual consumers’ neophobia reported by two of the dominant neophobia scales with their stated intent to buy/consume IoR products. In each case, the predictive value of the scale on behaviour was estimated by a straight correlation of one score against the other for the entire sample.

The first scale that was tested was the Food Technology Neophobia Scale (FTNS) [66]. This scale comprises 13 items, employing a Likert scale rating for assessment. The second scale was a more detailed food technology neophobia evaluation scale adapted from meat to fruit, consisting of 19 components [85].

To conclude the research, participants were prompted with a final question, asking them to identify the primary obstacle to adopting GMOs and IoR fruit, aiming to discern whether specific reasons, beyond neophobia as a general consumer psychological condition, held significance.

## 3. Results

### 3.1. Participant Profiles

The participant profiles are shown in Table 1, where the number of participants in the first replication (R1) is 159 and 154 in the second replication (R2). Most of the sample consisted of young participants; 74% under 25 years in R1 and 83% in R2. Over 80% were omnivores, with vegetarians being the second largest group; the average between the two replications is 10%. The sample had large groups of Caucasians (48% in R1 and 59% in R2) and Other (33% in R1 and 24% in R2), which makes cross-cultural comparisons impractical. However, as the sample was collected in a single country and city, it can be assumed that participants are generally exposed to the same fruit presentations in retail, such as supermarkets. As the research is not focused on preferences towards the fruit itself or consumption patterns, cultural differences may be minimal. The sample is a student sample and is demographically typical of a sample drawn from this particular university campus, with the exception for the low proportion of Māori (indigenous) students. The sample is thus capable of delivering a clear result with high internal validity due to its homogeneity.

Each replication has four different treatments (standard fresh fruit, GM fruit, IoR fruit, and fruit that has had both GM and IoR treatment). In the IoR group, less than 4% of the sample knew about IoR, and more than half claimed it was a completely new concept. In the GM group, 20% is the average between the two replications, where the sample responded that GM was a completely new concept.

### 3.2. RQ1—What Are Consumers’ Associations with GMOs and the IoR Concept?

The analysis resulted in the collection of 12 response types, outlined in Table 2. Various reactions falling within each of these types are also presented in the second column of Table 2. Responses that were un-codable included queries, irrelevant statements, and statements or terms with either no clear or ambiguous sentiment. A subsequent examination of these 11 groups revealed a clear distinction between the response types (1–4), predominantly affective and non-cognitive, and cognitive (5–11). These cognitive reactions delve into a more analytical and reasoned assessment of the perceived benefits and concerns associated with the concept, providing a nuanced understanding of participants’ viewpoints.

**Figure 1:**
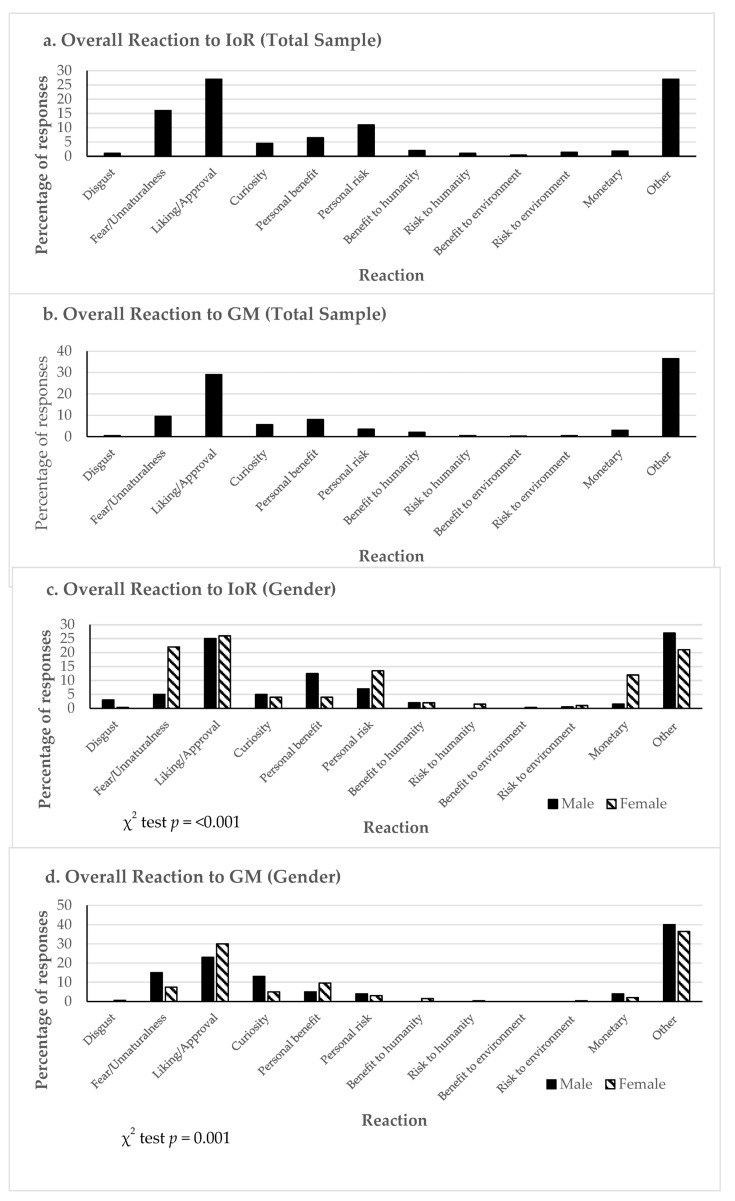

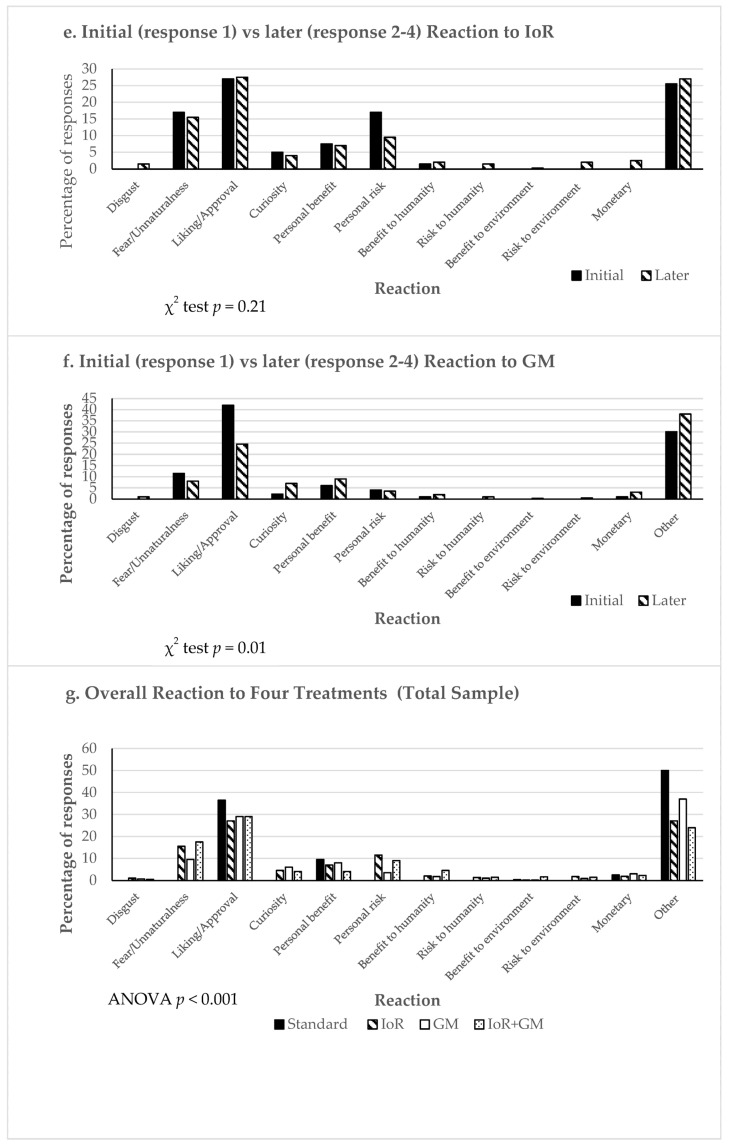
Sample Overall Reactions to concepts.

The frequency of these response types is illustrated in Figure 1, which comprises seven charts. The first two charts (Figure 1a,b) present the overall frequency of responses from both replications (R1 and R2) for IoR and GMOs, in which IoR shows a higher frequency of fear/unnaturalness and personal risk. The subsequent two charts (Figure 1c,d) break down the frequency based on gender. Conducting a Chi2 analysis to assess the significance of any variations in gender-related responses revealed no statistical significance. Figure 1e,f show the relative frequency of the groups for the initial response compared to the subsequent three of the four permitted responses. The relative frequency of the response type within the initial term provided by respondents in reaction to the IoR and GMO concept was then compared with the subsequent three responses they offered to determine if any “immediacy effect” could be identified. Although the Chi2 analysis indicated no significance, the histogram in Figure 1e,f shows a tendency for a higher proportion of non-cognitive/affective reactions in the initial response (reflecting heightened levels of fear/unnaturalness and liking/approval), except for the cognitive reaction related to IoR, specifically the personal risk aspect, which exhibits an increased frequency of responses.

The last chart (Figure 1g) indicates the overall frequency from R1 and R2 of free-text responses across the four treatments: GMIoR (GM and IoR), GM, IoR, and SF (standard fruit). While the category of liking/approval for IoR exhibits a high frequency of responses, it has the lowest overall frequency compared to the other three treatments. Likewise, the liking/approval category maintains a consistently high frequency of responses across all treatments. This indicates that consumers interpret the survey instructions for their reactions to the four treatments. Yet, they tend to streamline the complex information into a more familiar concept [103], resembling consumers’ positive responses when encountering a strawberry. Regarding cognitive reactions, once again, the frequency of responses is highest for the personal risk associated with IoR.

Apart from simply incorporating the response frequency shown in Figure 1, this research employs SAS Enterprise Miner for semantic analysis, as indicated in Table 3. Semantics are better suited to comprehending abstract concepts, such as emotional experiences—the use of text topic modelling as a semantic approach aids in identifying themes within textual datasets [104]. Relative and absolute frequencies of terms and topics within and between documents in each corpus are utilized to compute association weights. Terms identified in a specific document are assigned a weight indicating a certain association with a particular text topic, with a greater weight reflecting its significance. Based on the log-entropy weightage of the terms found in both R1 and R2, the IoR concept is more strongly associated with “radiation and chemical”, while the GMO concept is linked to “chemical”. This analysis suggests that consumers are more likely to equate IoR with radiation.

### 3.3. RQ2—What Are Consumers’ Behavioural Intentions towards GMOs and IoR Products?

**Figure 2:**
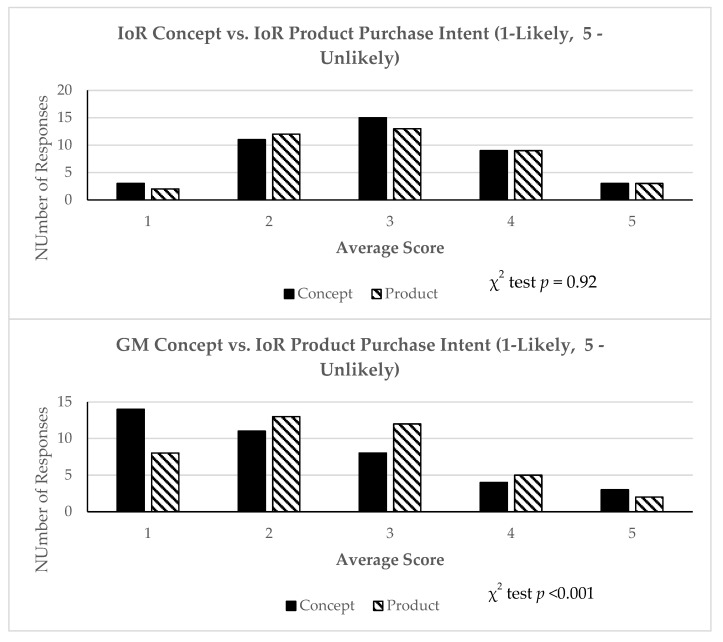
Response profile to GM and IoR concepts and products.

The findings from this portion of the study uncover substantial variations in the preferences for food technologies (Table 4, Table 5 and Table 6 and Figure 2). It has the lowest frequency of purchase intent when IoR was presented as a strawberry product (Table 4). This contrasts with the SF, which consumers will most likely purchase. On the other end of the scale, the GMIoR product has tzzhe most significant frequency in which consumers will not purchase. The unfavourable view of highly processed foods is primarily rooted in their perceived lack of naturalness [105].

Comparing the response profiles of IoR and GM (Figure 2) as concepts and when presented as products, it is evident that IoR responses tend to cluster around the neutral mean, while GM responses exhibit a significantly higher percentage of positive reactions (indicating a greater likelihood of purchase). The IoR concept and product illustrate a relatively similar response variance, whereas the GM concept shows a higher purchase probability than the GM product. This implies that the perception or idea of GM food conveyed by the concept was more positively received in terms of purchase intention than the specific GM product presented. It could indicate a disparity between the abstract notion of GM food and the tangible product regarding consumer acceptance or willingness to buy.

A significant *p*-value of the analysis of variance (ANOVA) typically indicates substantial differences among the four treatments, as shown in Table 5 and Table 6. In other words, the observed variations between the group means are unlikely to have occurred by random chance alone. In contrast to SF, there is a notable disparity in the response to IoR and GMIoR. This is evident from the considerably higher q Tukey score compared to the studentized range statistics. Nonetheless, the scenario shifts when contrasting SF with GM. While the q Tukey score is somewhat elevated, it does not reach the same level observed when IoR is introduced. This suggests that the presence of IoR has a distinctive impact on consumer response. In other words, consumers exhibit a greater acceptance of GM, in contrast to IoR, akin to the gradual overcoming of the initial reluctance seen with milk pasteurization.

### 3.4. RQ3—Does FTN Predict Consumer Attitudes and Behavioural Intentions towards IoR Effectively?

The association between technology neophobia, as indicated by an individual through FTNS, and the adapted consumer attitudes assessment scale [106] was examined about their declared intention to purchase IoR products. The results of these comparisons are presented in Table 7. The findings suggest that scores obtained from the FTNS exhibit a weak correlation with the intent to purchase (correlation of 0.34). This indicates a limited connection between the scores obtained from the FTNS and the stated intention of individuals to make a purchase. In other words, the scale may not strongly reflect consumers’ willingness to buy products associated with IoR. Additionally, key factors contributing to consumers’ reluctance to try foods created through novel food technologies typically encompass functional obstacles tied to the technology’s utility, perceptions of risk, the role of the media, and perceptions of healthiness [68]. As illustrated in Figure 1g, the personal risk factor associated with IoR has the highest frequency.

To further refine the analysis, correlations for the IoR FTNS were recalculated, including only the perception of risk elements from the FTNS (4 of the 13). This reduction in input information resulted in a slight decrease in the strength of the relationship between the FTNS and purchase intent, with a correlation of 0.24. This suggests that the perception of risk, as measured by FTNS, still plays a role in predicting consumer intentions to purchase IoR products, although to a lesser extent when compared to the broader FTNS measurement. Conversely, the adapted assessment scale by Bryant and Barnett [85] shows a relatively strong negative correlation to making a purchase, with a correlation of −0.70. This implies that participants who exhibit greater fear or resistance to novel technologies (higher neophobia scores) are more likely to express unfavourable attitudes towards IoR products, potentially leading to a decreased intention to purchase or adopt such technologies. In contrast to prior studies [87], the scale exhibited a robust association with the expressed intention to purchase (correlation 0.79), where individuals associate “clean meat” with traditional real meat. In this regard, striving to align IoR fruit with the characteristics of standard fresh fruit or normalizing the IoR treatment process as an everyday home appliance like a microwave (as one of the participants concurs, “radiation is weird, but I heat food with microwaves, so whatever”) is likely to enhance consumer acceptance.

## 4. Discussion

### 4.1. Research Implications

The analysis of the unstructured responses from the sample regarding IoR revealed that the response profile is predominantly affective, with fear/unnaturalness and liking/approval accounting for 43% of the 11 identified response types. These patterns exhibited minimal influence from gender, suggesting subconscious and non-cognitive reactions may guide them. Among the cognitive responses, personal risk holds the highest contribution at 11%. SAS text topic modelling is utilized to enhance the analysis; the result is in line with previous studies in demonstrating that consumers commonly associate IoR with radiation or radioactivity [8,16,24,25,29,32,50,51].

The implications of this research concerning the word association are threefold. First, the affect heuristic [80] suggests that individuals with more positive feelings towards strawberries are inclined to exhibit higher liking and approval, particularly towards the image of a strawberry depicted in the survey form. This tendency is observed independently of the underlying food technologies addressed in this research, with all participants receiving the same illustration of strawberries, but the instructions explicitly outlined the distinct treatments. Food consumers have been conditioned to respond to visual cues, and they might instinctively interpret the food label in a manner that influences their choices, even when this interpretation deviates from the label’s primary purpose of conveying detailed information [103]. Thus, marketers can capitalize on the influence of visual cues in shaping consumer decisions by integrating visually appealing design elements or employing images that elicit positive emotions and associations with the product.

Second, this study highlights the significance of terminology in communicating with consumers about novel food technologies. Stakeholders responsible for formulating guidelines and laws regarding the marketing and labelling of food products face the challenge of avoiding misleading consumers and ensuring that beneficial food innovations are not rejected due to unfavourable terminology. Individuals were more willing to consume CRISPR (Clustered Regularly Interspaced Short Palindromic Repeats) than GM food [36], possibly influenced by the innocuous acronym. Future research on naming, such as investigating consumers’ risk perceptions of acronyms like IRIS (Ionizing Radiation Is Safe), could offer interesting insights.

Lastly, events that occur frequently are generally more accessible for imagination and recall compared to rare events, leading people to depend on the availability heuristic as a mental strategy [84]. However, the memorability and imaginability of events are also influenced by various factors unrelated to their likelihood. As a result, this natural cognitive process causes individuals to overestimate the probabilities of recent or emotionally impactful events. Specifically, risks associated with radiation or radioactivity seem particularly prone to amplification by the availability heuristics, given the extensive media coverage they receive. The media’s emphasis on potential catastrophes contributes to the heightened risk perception, as fear sells [107]. Further, IoR treatment can be seen as relatively new regarding the communication of it to the end consumer of fruit and vegetable products. Achieving acceptance for IoR is a gradual process, requiring a well-established, long-term safety record, the involvement of a reputable and trusted regulatory authority, and a clear understanding of the associated benefits. Consumers tend to be more open to accepting increased risks when there are corresponding gains in benefits [108].

The final section of this study investigated the correlation between FTN and consumer attitudes towards IoR. The extensively used FTNS [66] was evaluated to ascertain if its scores significantly correlated with individual consumer purchase intent for IoR. The conclusive result indicated that they did not, deeming the instrument ineffective. Potential explanations include that the scale is positively linked to distrust in science and relies heavily on cognitive evaluation factors. As described earlier, consumers tend to stigmatize radioactivity more than investigate technological reliability, and their preferences align more with non-cognitive elements.

### 4.2. Future Research

There is a need to study consumers in their actual environments outside controlled experimental settings. It is recognized that real-world conditions, including social context, economic conditions, and cultural influences, can significantly influence consumer behaviour. The focus is on monitoring how consumers act in response to the availability of IoR products. The objective is to gain valuable insights into whether consumers’ perceptions match their behaviour. By comparing these perceptions with actual purchasing patterns, researchers can determine if there is consistency or disparity between what consumers say and what they do. Subsequently, assessing how consuming IoR foods may affect individuals’ long-term health and safety may improve consumers’ acceptance. This could involve monitoring various health indicators, conducting regular check-ups, and considering factors such as age, pre-existing health conditions, and lifestyle. Conducting a study such as this has the potential to provide evidence-based insights that can inform public health guidelines, regulatory decisions, and consumer choices. Ultimately, a study is needed that identifies which labelling strategies are most positively received by consumers. The objective is to assess how these strategies impact consumer acceptance, encompassing attitudes, perceptions, and willingness to purchase or consume IoR products. Understanding which labelling strategies resonate with consumers can enhance transparency, build trust, and potentially increase the acceptance of IoR foods in the market.

### 4.3. Limitations

This study has its limitations. IoR is still a hypothetical product, impacting the overall precision of consumer responses to the IoR concept compared to actual experiences with IoR products. The hypothetical purchase scenario also has the potential to introduce various biases, including the social desirability bias associated with consumers perceiving their behaviour as observed, the constrained exposure to a more limited choice set than typically available, and the absence of tangible consequences for a decision not involving an actual purchase. It is recognized that a comprehensive assessment of IoR products can only be conducted when the product is readily available [106]. Employing a sample of university students results in a homogenous participant pool that is younger and more educated and capable of delivering a research result with high internal validity. By the same logic, its capacity for extension onto the wider population requires further investigation. While this demographic may prefer novel concepts like IoR, it does not represent the broader population. For example, prior research suggests that younger, highly educated, and well-informed participants tend to exhibit more favourable attitudes towards IoR products [109].

## 5. Conclusions

The efficient management of highly perishable food supply chains, particularly for fruit and vegetables, is crucial due to rapid quality deterioration. This study focuses on IoR as a non-thermal technology for food preservation, emphasizing the need to understand consumer perceptions. The historical reluctance to adopt new food technologies, as illustrated by the past acceptance of pasteurization, sets the context for the current challenges faced by IoR. It highlights psychological factors influencing consumer acceptance, such as personality traits and FTN.

This research employed a survey with 313 participants at the University of Otago, using both closed and open-ended questions. The survey included treatments of standard fresh fruit, GM fruit, IoR fruit, and GM and IoR fruit. Participants engaged in word association tasks, and their responses were coded for analysis. This study investigated consumer reactions, associations, and willingness to purchase GMOs and IoR products. Text topic modelling was used to analyse emotional experiences related to purchasing these products. Neophobia, as a predictor of consumer attitudes towards IoR products, was assessed using established scales.

The responses to GMOs and IoR were analysed, revealing 11 reaction types. Analysing word associations revealed that emotional rather than cognitive factors mainly drove consumer responses to IoR. Furthermore, participants tend to link IoR with radiation or radioactivity. The study delves deeper into consumers’ readiness to consume these foods by evaluating the impact of the FTNS and attitudes. The FTNS showed limited predictive capability, while the 19-point adapted attitude scale demonstrated an inverse association between IoR neophobia and IoR fruit purchase intention. The influence of terminology on consumer perceptions was highlighted, emphasizing the need for clear communication when introducing novel food technologies.

## Figures and Tables

**Table 1:** Sociodemographic profiles.

Replication1 (R1) *n* = 159			
Gender		Race/Ethnicity	
Male	39 (25%)	European	76 (48%)
Female	118 (74%)	Other	53 (33%)
Other	2 (1%)	Asian	20 (13%)
		Chinese	9 (5.5%)
		Maori	1 (0.5%)
Age			
18 to 25 years	118 (74%)	Dietary practices	
26 to 50 years	35 (22%)	Omnivore	127 (80%)
51 to 65 years	5 (3%)	Vegetarian	18 (11%)
>65 years	1 (1%)	Other	10 (7%)
		Pescatarian	4 (2%)
			
Familiarity with IR (*n* = 40)		Familiarity with GM (*n* = 39)	
It was completely new to me	21 (53%)	It was completely new to me	8 (21%)
I’ve heard of it before	17 (43%)	I’ve heard of it before	26 (67%)
I felt well informed	2 (4%)	I felt well informed	5 (12%)
**Replication2 (R2) *n* = 154**			
Gender		Race/Ethnicity	
Male	52 (34%)	European	91 (59%)
Female	97 (63%)	Other	37 (24%)
Other	5 (3%)	Asian	15 (10%)
		Chinese	6 (4%)
		Maori	2 (1%)
Age		Prefer not to say	3 (2%)
18 to 25 years	128 (83%)		
26 to 50 years	24 (16%)	Dietary practices	
51 to 65 years	1 (0.5%)	Omnivore	133 (86%)
Prefer not to say	1 (0.5%)	Vegetarian	14 (9%)
		Other	6 (4%)
		Pescatarian	1 (1%)
			
Familiarity with IR (*n* = 38)		Familiarity with GM (*n* = 37)	
It was completely new to me	24 (63%)	It was completely new to me	7 (19%)
I’ve heard of it before	13 (34%)	I’ve heard of it before	25 (68%)
I felt well informed	1 (3%)	I felt well informed	5 (13%)

**Table 2:** Free text responses: considerable response categories.

Response Type	Examples
Non-cognitive/affective (IR)	
1—Disgust	“soggy; disrespectfulness; gross”
2—Fear/Unnaturalness	“scary; weird; not fresh; processed; skeptical; artificial; unnatural; fake; Modified; apprehension; concern; hesitate to eat”
3—Liking/Approval	“tasty; fresh; juicy; appealing; happy; innovative; necessary; futuristic; clean; yummy; smart idea; delicious; I want to eat; enjoyable”
4—Curiosity	“tempting; curious; fascinated, is it safe? is it healthier? Exploration; climate change? interesting”
Cognitive (IR)	
5—Personal benefit	“healthy; cheaper; flavor improved; vitamins; microbe-free; radiation is weird but I heat food with microwaves, so whatever”
6—Personal risk	“unhealthy; not knowledgeable; cancer; chemical; side effects; sickness; poison; unfamiliar; radioactivity; dangerous; contaminated”
7—Benefit to humanity	“mass production; food security; accessibility; less hunger”
8—Risk to humanity	“angry; harmful”
9—Benefit to the environment	“buy from the farmers market”
10—Risk to the environment	“radioactivity; wastage; non-organic”
11—Monetary	“cost; money; expensive”
12—Un-codable/other	“summer; red; sweet; colorful; sugar; supermarket; don’t judge the book by its cover; seeds; garden; tech plants; modern; pavlova; tart; berry; half eaten”
Non-cognitive/affective (GM)	
1—Disgust	“gross; vomit; choke”
2—Fear/Unnaturalness	“fake; artificial; suspicious; scary; unnatural; hesitate to eat; modified; weird; steroid; concern; uncertain; untrustworthy; unnecessary”
3—Liking/Approval	“useful; yummy; fresh; good; tasty; flavorful; cool; delicious; attractive; vibrant; enjoyable; smart; fun; futuristic; smell good”
4—Curiosity	“curious; interesting; will it taste fake? is it better? is it bigger/juicier? health impact? is it safe? is it healthy? odd texture; intriguing”
Cognitive (GM)	
5—Personal benefit	“healthy; more affordable; nutritional value; more conducive to health”
6—Personal risk	“unhealthy; safety concern; lack of understanding; chemical; toxic; rotten; allergic; sick; lower quality”
7—Benefit to humanity	“beneficial; potentially sustainable; improved”
8—Risk to humanity	“controversial technology”
9—Benefit to the environment	“good for the environment”
10—Risk to the environment	“ecological impacts; biosecurity”
11—Monetary	“luxury; money; expensive”
12—Un-codable/other	“summer; tropical; sweet; science; red; berry; ice cream; sour; smoothie; seeds; CRISPR; technology; consumerist; colorful; garden; milk; Christmas”

**Table 3:** SAS log-entropy frequency and term weight.

Concept	Responses Associated with Each Concept and Avoidance of Purchase
IR	“radiation, chemical, risk, health, effect, harmful, price”
GM	“chemical, harmful, modified, unnatural, effect, benefit, safe”

**Table 4:** Purchase intent for GMOs and IR products.

Purchase Intent	Concept		Strawberry Product		
	IR	IR	GM	GMIR	SF
1—Likely	3	2	8	5	23
2	11	12	13	14	12
3	15	13	12	10	5
4	9	9	5	7	1
5—Unlikely	3	3	2	5	0

**Table 5:** Analysis of variance (ANOVA) for the four treatments.

Source of Variation	*SS*	*df*	*MS*	*F*	*p*-Value	*F* Crit
Between groups	47.831	3	15.944.	14.790	**1.434 × 10^−7^**	2.664
Within groups	164.993	153	1.082			
Total	212.823	156				

**Table 6:** Tukey’s Honestly Significant Difference (HSD) test.

Group Pairs	Absolute Difference	Standard Error	*q* Tukey	*q* Table (5% Significance Level)
IR vs. GMIR	0.129	0.168	0.755	3.685
IR vs. GM	0.526	0.167	3.149	3.685
**IR vs. SF**	**1.388**	**0.170**	**8.136**	**3.685**
GMIR vs. GM	0.397	0.166	2.393	3.685
**GMIR vs. SF**	**1.260**	**0.166**	**7.586**	**3.685**
**GM vs. SF**	**0.862**	**0.166**	**5.193**	**3.685**

**Table 7:** Correlations between FTNS and the intention to purchase IR products.

FTNS	Elements	Correlation
(Cox & Evans, 2008) [66] food technology neophobia scale	**13**, **(1)** There are plenty of tasty foods around, so we don’t need to use new food technologies to produce more; **(2)** The benefits of new food technologies are often grossly overstated; **(3)** New food technologies decrease the natural quality of food; **(4)** There is no sense in trying out high-tech food products because the ones I eat are already good enough; **(5)** New foods are not healthier than traditional foods; **(6)** New food technologies are something I am uncertain about; **(7)** Society should not depend heavily on technology to solve its food problems; **(8)** New food technologies may have long-term negative environmental effects; **(9)** It can be risky to switch to new food technologies too quickly; **(10)** New food technologies are unlikely to have long-term negative health effects; **(11)** New products produced using new food technologies can help people have a balanced diet; **(12)** New food technologies give people more control over their food choices; **(13)** The media usually provides a balanced and unbiased view of new food technologies	**0.34** (IR strawberry purchase)
(Bryant & Barnett, 2019) [85] adapted assessment scale	**(19)**, **(1)** Eating Irradiated fruit is likely to be healthy; **(2)** Irradiated fruit is likely to look, taste, smell, and feel like the standard fruit you would find in your supermarket; **(3)** Irradiated fruit is likely to contain chemicals or ingredients which should be avoided; **(4)** Irradiated fruit is likely to be safe for human consumption; **(5)** I would trust Irradiated fruit; **(6)** Irradiated fruit is unnatural; **(7)** Irradiated fruit is appealing to me; **(8)** I feel positive about the development of Irradiated fruit; **(9)** The idea of Irradiated fruit is disgusting; **(10)** I feel comfortable about the idea of eating Irradiated fruit; **(11)** I would be anxious about eating Irradiated fruit; **(12)** Eating Irradiated fruit would conflict with my Values; **(13)** I feel that I would have control over my decision to eat Irradiated fruit or not; **(14)** Others would disapprove of me eating Irradiated fruit; **(15)** Irradiated fruit will have benefits for our society; **(16)** Production of Irradiated fruit is wise; **(17)** Production of Irradiated fruit is necessary; **(18)** Irradiated fruit is more environmentally friendly than the standard fruit you would find in your supermarket; **(19)** Producing Irradiated fruit poses a risk to society	**−0.70** (IR strawberry purchase)

## Data Availability

The data presented in this study are available on request from the corresponding author due to privacy/ethical restrictions.

## Appendix A. Survey Items

i. Word Association

Write up to four words, phrases, feelings, or thoughts that come to mind when you see this product (a smartphone).

The strawberry below is an irradiated fruit that has been treated with radiation to increase shelf-life and prevent rapid spoilage. Write up to four words, phrases, feelings, or thoughts that come to mind when you see this product.

	**Words, Phrases, Feelings, or Thoughts**	**Response Options**
Smartphone	___, ___, ___, ___	Highly positive (1)–Highly negative (5)
Irradiated fruit	___, ___, ___, ___	Highly positive (1)–Highly negative (5)

ii. How willing would you be to purchase irradiated fruit if it was available at the supermarket?

“I would definitely purchase” (1) to “I would definitely not purchase” (5)

iii. Attitude and behavioural intentions

**Table A1:** Response options for the quantitative measures (Bryant & Barnett, 2019) [85].

Measure	Items	Response Options
Attitude	Eating Irradiated fruit is likely to be healthy	Strongly agree (1)–Strongly disagree (5)
	Irradiated fruit is likely to look, taste, smell, and feel like the standard fruit you would find in your supermarket	
	Irradiated fruit is likely to contain chemicals or ingredients which should be avoided	
	Irradiated fruit is likely to be safe for human consumption	
	I would trust Irradiated fruit	
	Irradiated fruit is unnatural	
	Irradiated fruit is appealing to me	
	I feel positive about the development of Irradiated fruit	
	The idea of Irradiated fruit is disgusting	
	I feel comfortable about the idea of eating Irradiated fruit	
	I would be anxious about eating Irradiated fruit	
	Eating Irradiated fruit would conflict with my values	
	I feel that I would have control over my decision to eat Irradiated fruit or not	
	Others would disapprove of me eating Irradiated fruit	
	Irradiated fruit will have benefits for our society	
	Production of Irradiated fruit is wise	
	Production of Irradiated fruit is necessary	
	Irradiated fruit is more environmentally friendly than the standard fruit you would find in your supermarket	
	Producing Irradiated fruit poses a risk to society	

iv. What would most prevent you from purchasing irradiated fruit?
v. Food Technology Neophobia Scale (FTNS)

**Table A2:** Response options for FTNS (Cox & Evans, 2008) [66].

Items	Response Options
There are plenty of tasty foods around, so we don’t need to use new food technologies to produce more	Strongly agree (1)–Strongly disagree (5)
The benefits of new food technologies are often grossly overstated	
New food technologies decrease the natural quality of food	
There is no sense in trying out high-tech food products because the ones I eat are already good enough	
New foods are not healthier than traditional foods	
New food technologies are something I am uncertain about	
Society should not depend heavily on technology to solve its food problems	
New food technologies may have long-term negative environmental effects	
It can be risky to switch to new food technologies too quickly	
New food technologies are unlikely to have long-term negative health effects	
New products produced using new food technologies can help people have a balanced diet	
New food technologies give people more control over their food choices	
The media usually provides a balanced and unbiased view of new food technologies	

vi. Self-rated familiarity with irradiated fruit:

“Which of the following most closely represents your prior exposure to Irradiated fruit before this study?”

- Completely new to me;
- I’ve heard of it before;
- I am well informed.
